# Epidemiology of Anosmia in South Korea: A Nationwide Population-Based Study

**DOI:** 10.1038/s41598-020-60678-z

**Published:** 2020-02-28

**Authors:** Jeong Wook Kang, Young Chan Lee, Kyungdo Han, Sung Wan Kim, Kun Hee Lee

**Affiliations:** 1 Department of Otolaryngology-Head and Neck Surgery, Kyung Hee University School of Medicine, Seoul, Republic of Korea; 2 Department of Biostatics, Catholic University College of Medicine, Seoul, Republic of Korea

**Keywords:** Health policy, Epidemiology

## Abstract

Research about the epidemiology of olfactory dysfunction in Asians was not enough. The purpose of this study was to assess the prevalence and incidence rate of olfactory disorders in Koreans and to evaluate demographic risk factors. We analyzed clinical data of patients with anosmia using Korean National Health Insurance Service data from 2006 to 2016. The data includes medical insurance claim data and medical records of almost 50,000,000 people in Korea. The 30-39 age group showed the highest prevalence (19.25 per 10,000 per year). Their incidence rate was also high comparing other age groups (13.30 per 10,000 per year). The prevalence and the incidence increased from 7.10 to 13.74 and from 5.66 to 9.54 between 2006 and 2016. In the seasonal analysis, the incidence rate was high in spring and autumn. The high-income population showed about 1.4-folds higher incidence rate than the low-income population. We thought that the socioeconomic status could generally affect the rate of hospital visit in the anosmia population. Anosmia can be frequently underdiagnosed in the clinical environment because the elderly and the low-income people easily underestimate their anosmia symptom and ignore the severity due to their economic problem. Therefore careful attention and further studies for anosmia are needed.

## Introduction

Olfaction is the sense of smell that chemically detects odorants in the air. Perhaps the most crucial function of olfaction is an early warning system for the detection of fire, leaking of natural gas, contaminated air, and spoiled food^1–4^. It also has functions to determine the flavor and palatability of foods and drinks^1,4^. Despite many roles of olfaction, it is regarded less importantly compared to vision or hearing^1^. Another important reason why the importance of olfactory disorder is overlooked is that we cannot easily detect olfactory depletion^1,5^. Young patients with congenital olfactory disorders show tend not to aware of olfactory disturbances of their own^5^.

So far, there is no standardized diagnostic method for anosmia. Although olfactory event-related potentials (OERPs) and electro-olfactogram (EOG) can be used to objectively measure olfactory function, they have many limitations to be used as an objective diagnostic method until now. For these reasons, psychophysical tests are quite commonly used, such as the University of Pennsylvania Smell Identification Test (UPSIT) or Sniffin’ Sticks^6–9^. UPSIT was developed by American and Sniffin’s Sticks test was invented by Germany G. Kobal. UPSIT contains odors in 40 microencapsulations, which are then scratched with a pen and sniffed to identify odors, then identifying the olfactory status by percentiles according to age and gender. UPSIT, however, has a lot of smells that are not familiar to Koreans, and because of the one-time use, it is expensive and involves only an identification test. Therefore, in Korea, Korean Version of Sniffin’ Sticks Test (KVSS), which is inexpensive and can be repeatedly conducted, has been developed and used for the diagnosis of anosmia.

Over the past decade, we have rediscovered clinical implications of olfactory loss due to advances in diagnostic technology and improving lifestyle. The relationship between olfactory dysfunction and neurodegenerative disorders such as mild cognitive impairment, Alzheimer’s disease, Parkinson’s disease, frontotemporal dementia, vascular dementia, and multiple sclerosis is now well-established. Many study groups have proposed that olfaction may be a useful biomarker for neurodegenerative diseases, showing that olfaction can be used for early precede disease diagnosis, differential diagnosis, and prediction of clinical outcomes in neurodegenerative diseases^10–15^. Recent studies have shown that olfaction also affects the quality of life (QOL)^1,5,16–19^. Olfactory impairment can lead to problems in diverse elements of QOL such as safety, hygiene, and nutrition.

Nevertheless, unlike vision and hearing, there is no international standard to test olfactory function. Different types of olfactory tests are being conducted in each country and region. The fact that there is no standardized method makes studying olfactory more difficult. Although epidemiology is the basis of disease investigation, few large scale epidemiologic studies of olfactory function have been reported. Although some studies on olfactory dysfunction have been carried out in several countries, the prevalence in the general population in Asia remains unclear^2,20– 22^. Thus, the objective of this study was to investigate the prevalence, incidence rate, and risk factors in Korea to improve our knowledge of olfactory disorders.

## Results

### Prevalence and incidence rates according to age group

**Figure 1:**
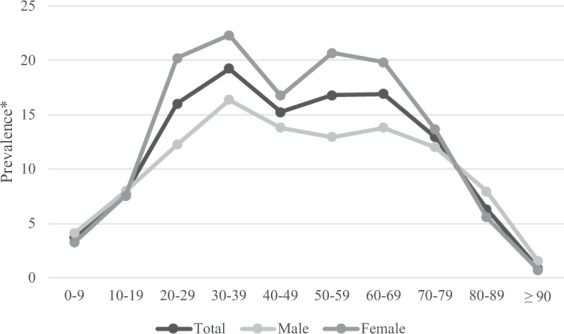
Prevalence of anosmia patients in 2015.

**Figure 2:**
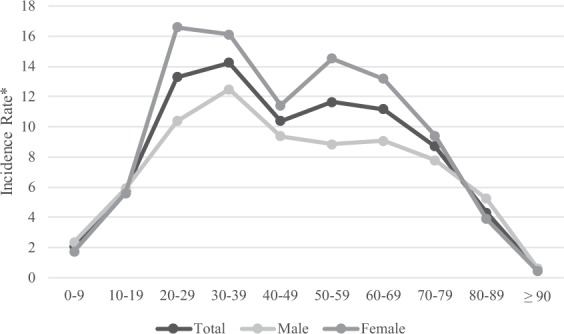
Incidence rates of anosmia patients in 2015.

Analysis of the prevalence of anosmia patients in each age group based on the customized database (DB) in 2015 showed that the prevalence of anosmia in women 20–79 years old was relatively higher than that in men (Fig. 1, Table 1). The prevalence of anosmia was the highest at 19.25 per 10,000 in males and females in 30–39 years old. The prevalence of anosmia was 16.25 per 10,000 in males in 30–39 years old and 22.28 per 10,000 in females in 30–39 years old. The prevalence of anosmia in males was maintained after 30-39 years old and then decreased after 70–79 years old, but the prevalence of anosmia in females increased again after 50–59 years old and showed a double peak pattern. The prevalence of anosmia in those  < 10 years old and ≥90 years old were under 5 per 10,000. The incidence rate of anosmia in females was higher than that in males aged 20–70 in 2015 (Figure 2, Table 2). The incidence rate of anosmia in males was the highest at 12.48 per 10,000 in 30–39 years old and that in females was the highest at 16.57 per 10,000 in 20–29 years old. After 30–39 years old, the incidence rate of anosmia in males was maintained and then decreased. Females showed a peak of incidence rate in 30–39 years old and a repeat increase in 50-59 years old. The incidence rate of those aged <10 and ≥80 years old was lower than 5 per 10,000. Prevalence and incidence rate of anosmia were similar between males and females at all age groups and the difference between prevalence and incidence rate was less than 3.37 per 10,000.

**Table 1:** Prevalence of anosmia patients in 2015.

Age	Population	Total	Prevalence^*^	Population	Patients^a^	Male	Population	Patients^a^	Female
		Patients^a^				Prevalence^*^			Prevalence^*^
0–9	4,605,930	1,698	3.68655	2,370,685	973	4.10430	2,235,245	725	3.24349
10–19	5,980,471	4,653	7.78032	3,126,473	2,495	7.98024	2,853,998	2,158	7.56132
20–29	6,821,889	10,916	16.00143	3,602,702	4,406	12.22971	3,219,187	6,510	20.22250
30–39	7,945,906	15,296	19.25016	4,074,241	6,669	16.36869	3,871,665	8,627	22.28240
40–49	8,934,467	13,614	15.23762	4,535,856	6,250	13.77910	4,398,611	7,364	16.74165
50–59	8,210,181	13,795	16.80231	4,121,285	5,339	12.95470	4,088,896	8,456	20.68040
60–69	4,691,566	7,925	16.89201	2,276,167	3,141	13.79951	2,415,399	4,784	19.80625
70–79	3,124,667	4,045	12.94538	1,327,943	1,593	11.99600	1,796,724	2,452	13.64706
80–89	1,110,631	700	6.30272	345,069	274	7.94044	765,562	426	5.56454
≥90	148,336	13	0.87639	33,098	5	1.51067	115,238	8	0.69422

^a^Number of patients with anosmia. ^*^Per 10,000, per year.

**Table 2:** Incidence rates of anomia patients in 2015.

Age	Population	Total	IR^*^	Population	Male	IR^*^	Population	Female	IR^*^
		Patients^a^			Patients^a^			Patients^a^	
0–9	4,602,076	946	2.05559	2,368,510	559	2.36013	2,233,566	387	1.73266
10–19	5,964,240	3,437	5.76268	3,117,499	1,840	5.90217	2,846,741	1,597	5.60992
20–29	6,780,510	9,019	13.30136	3,583,367	3,720	10.38130	3,197,143	5,299	16.57417
30–39	7,877,226	11,222	14.24613	4,048,262	5,054	12.48437	3,828,964	6,168	16.10880
40–49	8,848,771	9,189	10.38449	4,501,972	4,225	9.38478	4,346,799	4,964	11.41990
50–59	8,122,758	9,465	11.65245	4,088,967	3,614	8.83842	4,033,791	5,851	14.50497
60–69	4,634,873	5,176	11.16751	2,256,797	2,044	9.05708	2,378,076	3,132	13.17031
70–79	3,092,156	2,693	8.70913	1,316,845	1,025	7.78376	1,775,311	1,668	9.39554
80–89	1,103,176	475	4.30575	342,734	179	5.22271	760,442	296	3.89247
≥90	147,895	7	0.47331	32,951	2	0.60696	114,944	5	0.43499

^a^Number of patients with anosmia. ^*^IR, incidence rate (per 10,000, per year).

### Prevalence and incidence rate according to annual and seasonal change

**Figure 3:**
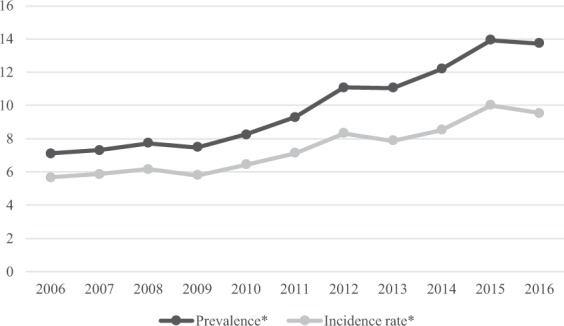
Age-sex standardized prevalence and incidence rate of anosmia by year.

**Figure 4:**
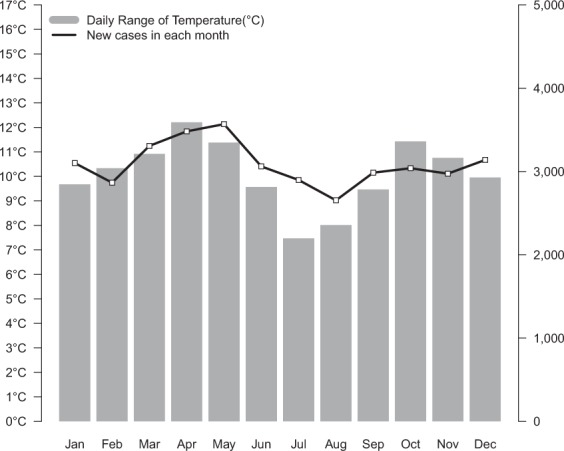
Comparison between incidence and seasonal daily range of temperature.

  Figure 3 shows the number of patients who have anosmia code as a major or minor disease in each corresponding year in the customized DB. Age-sex standardized prevalence and incidence rate maintained between 2006 and 2009. They have been increasing since 2009. Between 2006 and 2016, the prevalence changed from 7.10 to 13.74 per 10,000. The incidence rate changed from 5.66 to 9.54 per 10,000. Compared with 2006 data, the age-adjusted prevalence increased by 1.9-fold and the incidence rate increased by 1.7-fold in 2016. The difference between prevalence and incidence rates increased from 1.4 per 10,000 in 2006 to 4.2 per 10,000 in 2016. Figure 4 shows annual changes in overall incidence of anosmia. The overall incidence was the highest at 3568 patients in May and the lowest at 2653 patients in August. It has the second highest annual incidence in December and January. To analyze the relationship between overall incidence and seasonal climate, we used climate data from the Korea Meteorological Administration. We calculated the ‘daily range of temperature’ of 72 major cities by subtracting minimum temperatures from maximum temperatures of days. Figure 4 shows the change of annual ‘daily range of temperature’ variation in Korea. ‘Daily range of temperature’ in Korea, a country with mid-latitudes, is large in March-May period of spring and in September-November period of autumn. In a linear regression analysis, the overall incidence rate of anosmia and daily range of temperature were positively correlated (r^2^ = 0.494, p-value = 0.006).

### Prevalence and incidence rate according to the general characteristics

**Figure 5:**
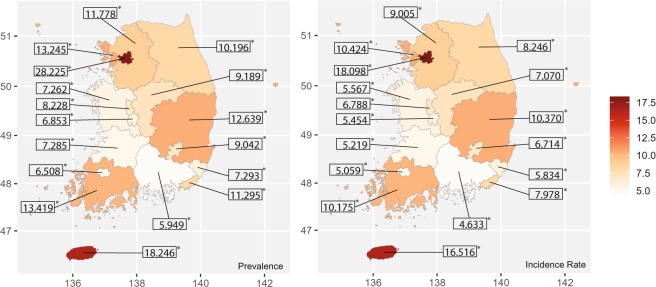
Age-sex standardized prevalence(left) and incidence rates(right) according to administrative district in 2015.

**Figure 6:**
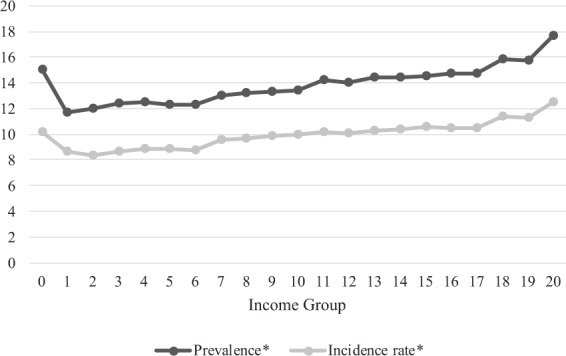
Prevalence and incidence rate of anosmia according to income group in 2015.

  Figure 5 shows age-sex standardized prevalence and incidence rates of anosmia according to their residence in 2015. The age-sex standardized prevalence of anosmia was the highest at 28.23 per 10,000 in Seoul, followed by that in Jeju at 18.25 per 10,000. Except for Seoul and Jeju, prevalence in other regions was similar. Gyeong-sang-nam-do had the lowest prevalence at 5.94 per 10,000. The pattern of incidence rate was similar to that of the prevalence. The incidence rate was 18.1 per 10,000 in Seoul and 16.52 per 10,000 in Jeju. Gyeong-sang-nam-do showed the lowest incidence rate at 4.63 per 10,000. The difference between prevalence and incidence rate was the largest at 10.13 per 10,000 in Seoul. It was not such large in Jeju, although prevalence and incidence in Jeju were high. Figure 6 shows age-sex standardized prevalence and incidence rates by income level in 2015. The prevalence and incidence rate of anosmia increased according to income level. The lowest income earners had exceptional high prevalence and incidence rates. The prevalence of anosmia according to the general characteristics of the participants is shown in Table 3. The age-sex matched chi-square analyses showed that residence, income status, and hypertension statistically related to the risk of anosmia.

**Table 3:** General characteristics according to the presence of anosmia.

Characteristics	Anosmia (R430)	Yes	P-value
	No		
	(n = 515,904)	(n = 171,968)	
Sex(male)	200,961 (38.95%)	66,987 (38.95%)	1
Age groups			1
20–39	204,561 (39.65%)	68,187 (39.65%)	
40–59	220,731 (42.79%)	73,577 (42.79%)	
60–79	86,901 (16.84%)	28,967 (16.84%)	
≥80	3,711 (0.72%)	1,237 (0.72%)	
Residence (Rural)	274,300 (53.17%)	73,206 (42.57%)	<0.0001
Low income^*^	146,168 (28.33%)	44,884 (26.10%)	<0.0001
Diabetes mellitus	34,848 (6.75%)	11,683 (6.79%)	0.5774
Hypertension	95,331 (18.48%)	33,867 (19.68%)	<0.0001
Dyslipidemia	76,545 (14.48%)	30,925 (17.98%)	<0.0001

^*^Income level ≤5.

## Discussion

Although the anosmia(R430) code which used in this study is usually applied by an otolaryngologist, other doctors can apply the code too in Korea. Otolaryngologist would get training on how to apply the code. But, authors cannot assure whether other doctors underwent related training about olfaction tests via a specific seminar. Authors guess that most of the cases applying the code are when a patient seeking medical care for anosmia or being diagnosed after an olfaction test such as KVSS.

Anosmia is not a final diagnostic name that describes the cause of olfactory impairment. There are many factors that can cause anosmia, including cumulative damage to the olfactory epithelium, damage to olfactory receptor, change of central olfactory pathway, activity change in neurotransmitters, altered nasal engorgement, increased propensity for nasal disease, decrements in mucosal metabolizing enzymes, ossification of cribriform plate foramina, and neurodegenerative disease^4,10,23–27^. Although the degree of impairment of olfactory function may not be directly correlated with the degree of olfactory neuron depletion, many of those factors appear to be worsen with age. Thus, it is widely accepted that the olfactory function will decrease with aging. This theory has been supported by some cross-sectional studies showing that almost half of the general population 65–80 years old have verifiable smell loss and that approximately three-quarters of the population over 80 years have such loss^10,20,22,28^. Interestingly, in this study, despite of low performance of olfaction in the elderly, their prevalence and incidence rates were relatively low rather than high (Figs. 1 and 2; Tables 1 and 2). Murphy *et al*. have suggested that the self-report system can significantly underestimate the prevalence than that obtained by olfaction test^20^. This suggests that elderly persons tend not to be aware of their olfactory impairment. But according to Lee *et al*. study that accepted self-reporting diagnosis, it seems that the elderly participants of the 2009 Korea National Health and Nutrition Examination Survey (KNHANES) could recognize their olfactory impairment well^21^. It means that elderly people could be aware of olfactory impairment, but they do not want to seek treatment. Moreover, hyposmia patients paradoxically tend to mention more complaints than anosmia patients^5^. As Lee *et al*. study, traditional study accepted self-reporting diagnosis on general population. This study also counted self-reported olfactory dysfunction but the olfactory dysfunction reported by people who are actively seeking treatment for it. We should consider this point as a cause of the large discrepancy of numbers between traditional study and our study. For these reasons, the younger and older population of this study would show lower prevalence and incidence than that of traditional study (Figs. 1 and 2; Tables 1 and 2). The authors thought that the double peak pattern of the incidence in female could be affected by postmenopausal olfactory dysfunction^29^.

Women in all ages, including adolescents, have a better olfactory function than men^28^. It is still unclear which causes make the gender difference. Similar gender differences have also been found in visual, auditory, tactile sense, and kinesthesis. Although female usually have better olfactory function, the prevalence of anosmia in females was high in this study. This might be due to the fact that women are more sensitive to olfactory dysfunction (Figs. 1 and 2; Tables 1 and 2)^5^.

Recently, as interest in quality of life and accessibility to medical care are getting higher, the number of patients with olfactory disorder who are looking for hospitals seems to be increasing. As a result, the prevalence of olfactory disorder has been increasing continuously since 2006. This might be due to increased diagnosis rate rather than increased incidence rate.

Unlike other chronic diseases, difference between prevalence and incidence rate of anosmia is small. This can be explained by the fact that major causes of anosmia would be diseases having a short recovery time. Common causes of olfactory disturbance have been known as upper respiratory infection (URI), chronic nasal and paranasal sinus disease (SND), and head trauma^2,3,5^. In fact, these three things account for about 90% of the cause in olfactory dysfunction and the sum of URI and SND portions exceeds 50%^5^. Because URI and SND treatment periods are relatively short, they could serve as a reason for narrowing the difference between prevalence and incidence rate of anosmia.

It is better to directly compare between URI incidence and the anosmia incidence in an analysis of seasonal changes. However, the authors could not access the incidence of URI because of the authorization limit. So, we replaced that with ‘daily range of temperature’. In Korea, spring is from March to May, summer is from June to August, autumn is from September to November, and winter is from December to February. Due to the climatic nature, the ‘daily range of temperature’ is large in spring and fall and the incidence rate of URI is increased in this period (Fig. 4). A similar pattern of new cases in each month could be a result of these climatic characteristics (Fig. 4).

Variation of disease incidence by region can be caused by differences in population structure, climate, and socio-cultural and economic features. When analyzing the high incidence of anosmia in Seoul and Jeju, it is reasonable to assume that these two cities have different causes due to their differences in climate, geologic location, and socioeconomic features (Fig. 5). Seoul is the capital located in the northern part of Korea with high-income level and many large hospitals. If income levels are high, it is common for individuals to access easily to medical care^20,21^. Therefore, the high incidence rate of Seoul could be due to these advantages in medical accessibility. It has been reported that air pollution could affect smell perception^4,14^. Seoul has high air pollution. Most citizens of Seoul frequently use the subway which is contaminated with fine dust. These factors must not be ignored because they have a long-term influence on olfactory function. Jeju is a volcanic island that many tourists visit. Jeju has a warm climate and many huge mandarin orange orchards. With warm climate, Japanese cedar is very abundant in Jeju. Some people of Jeju suffer from severe allergic symptom in springtime^30^. Leaf mite, an important cause of occupational allergic rhinitis in farmers working in the orchard, might have affected the incidence of anosmia^31,32^. In addition, mandarin orange orchards have relatively high pesticide usage per unit area compared to that of other fruits. Some papers have suggested that exposure to herbicides and insecticides can affect the olfactory function^10,19,33^. These are just hypotheses. Further study will be needed to clarify this issue.

In previous studies, low-income is associated with olfactory dysfunction^20,21^. High-earners can easily access health care service. They also have healthy lifestyles. Consequently, they are more likely to have relatively good health status. As a result, the low-income population tends to have higher prevalence of olfactory dysfunction than high-income earners. However, our results showed that high-income earners had higher incidence rate of anosmia than low-income earners (Fig. 6). When we interpret this result, we should consider that our study was based on the ‘customized DB’ with data collected by a self-report system. The self-reporting rate could be easily affected by socioeconomic status. Table 3 revealed the paradoxical aspect that low income related to the normosmic condition. In Fig. 6, the minimum income group showed an exceptionally high incidence rate compared to general low-income groups. The lowest-income group receives ‘Medical Aid’ benefits from the Korean government, meaning they can get medical care without payment. Their medical accessibility was greatly increased by ‘Medical Aid’. Their high incidence could be affected by these benefits.

It is unclear whether this study showed actual prevalence and incidence rate of olfactory disorder because it used data based on disease code collected from patients who visited hospital themselves having a complaint of olfactory dysfunction. Compared with other studies, the lower prevalence and incidence rate of our study in the elderly might be caused by such reasons. Another limitation of this study was that the diagnostic criteria of anosmia were not standardized in each hospital. The claim data didn’t include how they diagnose anosmia. The authors examined several risk factors for anosmia such as age, sex, average daily temperature change, geographical location, and income level. However, we could not present the statistical significance because of the authorization limit and imperfection of NHISS database itself.

Nevertheless, this study revealed reliable prevalence, incidence rate, and some risk factors of olfactory disorder in Asian people. We used the Korean National Health Insurance Service (KNHIS) Claims Database which contained all claims data for the Korean National Health Insurance program and the Korean Medical Aid program. The KNHIS covers almost 99% of the Korean population. It means that our study is near to a whole population study than a sample study. We also found a possibility that the socioeconomic status could affect the rate of hospital visitation in the general population. Further studies are needed to analyze the potential mechanisms underlying the association between socioeconomic status and anosmia incidence.

## Methods

The Korean National Health Insurance Service (KNHIS) is the public medical insurance system which is operated by the Ministry for Health, Welfare, and Family Affairs^34^. As a compulsory social insurance system, the KNHIS program covers about 97% of the entire Korean population. The remaining 3% of the population are covered by a Medical Aid program. The KNHIS database includes patient demographics and records on diagnosis, interventions, and prescriptions^35^. The diagnostic system of the KNHIS database was developed using the International Classification of Disease, Tenth Revision, Clinical Modification (ICD-10-CM) codes. Anosmia was defined as R430.

The KNHIS has been operating National Health Insurance Sharing Service(NHISS) to support academic research by utilizing National Health information (https://nhiss.nhis.or.kr/bd/ab/bdaba000eng.do). The NHISS provide some kinds of research database (DB), including ‘Customized DB’. The customized DB includes medical insurance claim data and medical records of almost 50,000,000 people who are medical aid beneficiaries and health insurance beneficiaries in Korea. We analyzed the customized DB which included most Koreans and some foreigners who had lived in Korea from 2006 to 2016. Target patients were defined as those whose anosmia was confirmed by a primary or secondary diagnostic name at a medical institution. The diagnosis of olfactory disorder was confirmed using the diagnostic code (R430, anosmia) in the health insurance claim data. Longitudinal analyses were performed using the data from 2006 to 2016. Cross-sectional analyses were conducted using data of 2015 which was relatively recent with sufficient quality. The income level group was obtained by classifying the population into 21 quartiles based on Korean health insurance premiums. A higher group means higher income.

This study was approved by the Institutional Review Board of Kyung Hee University Hospital at Gangdong (Approval No. KHNMC 2018-05-012) and Korean National Health Insurance Sharing Service (Approval No. NHIS-2018-1-242). Informed consent requirement was waived by the Institutional Review Board of Kyung Hee University Hospital at Gangdong because personal identifying information was not accessed. All research was performed in accordance with relevant guidelines and regulations.

Statistical analyses were performed using SAS software version 9.4 (SAS Institute, Cary, NC, US). Map chart was drawn using R Project for Statistical Computing version 3.5.3 (http://www.r-project.org). The prevalence and incidence rate of anosmia were calculated by dividing the number of cases by 10,000 person-years. Age adjusted prevalence and incidence rate were used for analysis. The chi-square test was used to compare the characteristics between participants diagnosed with anosmia and others being normosmia. The two groups were selected by randomly matching the age and sex of the patient group in the entire population.
